# Long-Term Functional Results of a Modified Caudal-to-Cranial Approach in Laparoscopic Segmental Left Colectomy for Diverticular Disease

**DOI:** 10.1155/2021/8940682

**Published:** 2021-01-11

**Authors:** Michele Manigrasso, Marcella Pesce, Marco Milone, Pietro Anoldo, Anna D'Amore, Giovanni Galasso, Nicola Gennarelli, Francesco Maione, Sara Vertaldi, Giovanni Sarnelli, Giovanni Domenico De Palma

**Affiliations:** ^1^Department of Advanced Biomedical Sciences, “Federico II” University of Naples, Via Pansini 5, 80131 Naples, Italy; ^2^Department of Clinical Medicine and Surgery, “Federico II” University of Naples, Via Pansini 5, 80131 Naples, Italy; ^3^Operative Unit of Gastroenterology, Pineta Grande Hospital, Via Domitiana Km 30, 81030 Castel Volturno, Caserta (CE), Italy

## Abstract

A modified caudal-to-cranial approach to perform laparoscopic left colectomy for benign diseases has been recently designed to facilitate the low-tie mesenteric dissection. A chart review has been performed including all consecutive patients with uncomplicated diverticulitis who have been treated by segmental left colectomy with a caudal-to-cranial approach. A total of 34 patients were included in the study. 21 patients were male, mean age was 54.1 ± 11.3, and mean BMI was 26 ± 5.5. Patients with ASA Score I were 7, with ASA II were 9, and with ASA Score III were 5. Incontinence Score (IS) resulted in an average of 5 ± 2, 2 grade of incontinence and the CS score showed an average of 10 ± 3, 2 grade of constipation. Health status, evaluated by Short Form-36 questionnaire, was demonstrated in these patients' great physical function, role, general health, and social function. The anorectal manometry performed 6 months after surgery showed a normal value in terms of the anal resting pressure (47 ± 13 mmHg) and an increased volume to stimulate desire to defecate (197 ± 25 ml). The length of the anal sphincter was normal compared to the reference value (37 ± 5.4 mm). Although further studies are required to obtain definitive conclusions, our results are encouraging to propose low-tie segmental colectomy as the standard procedure for the treatment of uncomplicated diverticulitis, and our modified surgical approach could be considered useful to facilitate the surgical approach.

## 1. Introduction

Diverticular disease (DD) represents the fifth most important gastrointestinal disease in Western countries with an estimated mortality of 2.5/100000 per year [1]. Although surgical treatment remains the gold standard in the case of complicated diverticulitis (Hinchey Classification Stage III and IV), the optimal management for treating uncomplicated diverticulitis is still under debate.

Previous guidelines recommended to undergo surgery for the patients after two attacks of acute diverticulitis.

Recently, new guidelines recommend to consider surgery on a “case-by-case basis,” according to patients' quality of life [2].

Anyway, the surgical treatment of choice to approach uncomplicated diverticular disease remains laparoscopic sigmoidectomy.

A modified caudal-to-cranial approach to perform laparoscopic left colectomy for benign diseases has been recently designed to facilitate the low-tie mesenteric dissection [3]. The mesenteric resection close to the colonic wall has been demonstrated to guarantee better functional results, i.e., reduction of the incidence of defecatory disorders and less lifestyle alterations, even if additional technical challenges have been described.

Personal experience on short- and long-term outcomes of this modified approach has been described to evaluate the safety and efficacy of the caudal-to-cranial approach.

## 2. Materials and Methods

A chart review has been performed including all consecutive patients with uncomplicated diverticulitis, from September 2013 to December 2018.

All patients affected by uncomplicated diverticulitis (Hinchey I/II) following successful medical treatment (4–6 weeks from surgery) were included in the study. The presence of diverticulitis was assessed by colonoscopy and TC of the abdomen [4].

Patients undergone to previous intervention that could have modified the pelvic nerve pattern (hysteroannessiectomy, colorectal surgery, etc.) were excluded from the study.

Perioperative prophylaxis routinely included subcutaneous heparin administration and perioperative handling of antiplatelet drugs, according to the current literature [5, 6]. Standard bowel preparation was given to all patients. The procedure was performed under general anesthesia.

Surgical intervention has been performed in all cases by a minimally invasive procedure, with a modified caudal-to-cranial approach as previously described [1, 7].

In detail, the intervention began with the creation of a passage into the mesentery of the upper rectum, followed by the positioning of a linear stapler and the section of the rectum. Then, a modified caudal-to-cranial dissection of the mesentery was performed towards the proximal healthy descending colon. Then, the splenic flexure was mobilized by a caudal-to-cranial dissection of the left parietocolic gutter.

Finally, a colorectal end-to-end anastomosis according Knight-Griffen technique was performed.

Perioperative management has been organized according to a standardized ERAS perioperative care protocol [8]. In detail, the nasogastric tube was removed at the end of the surgical procedure, and a free fluid oral intake was allowed 6 hours after surgery and a semisolid diet during the same day. Early mobilization was encouraged, and the urinary catheter was removed one day after surgery. Discharge criteria included the absence of symptoms, the tolerance to a semisolid diet, and the passage of stools.

The presence of defecatory disorder was evaluated, 6 months after surgery, by Ano-Rectal Manometry (ARM) and by the Jorge-Wexner Incontinence Score (IS) [9] and the Agachan-Wexner Constipation Score (CS) [10]. The IS evaluates the presence of incontinence of solid, liquid, or gas as well as the need to wear a perineal pad and daily lifestyle alterations. The score ranges from 0 (full continence) to 20 (total incontinence). The CS score evaluates the frequency of bowel movements, stool consistency, feeling of incomplete evacuation, difficulty, number of failure, and time spent to evacuate, need to use laxative, or manual help to defecate. The scale is from 0 to 30 points, and it represents the entity of the constipation. Furthermore, we used the Standard Medical Outcomes Survey Short Form 36 validated Italian Version (SF-36) to assess health status and the impact of defecatory disorder on normal life habits [11].

### 2.1. Data Assessment and Outcomes Evaluation

Data collected included age, gender, BMI, ASA score, and Hinchey Score.

The analyzed outcomes were the Jorge-Wexner Incontinence Score (IS), the Agachan-Wexner Constipation Score (CS), the Anal resting pressure, the threshold for desire to defecate, and the length of the anal sphincter.

### 2.2. Statistical Analysis

Statistical analysis was performed with SPSS 23.0. (SPSS Inc., IBM, Armonk, NY, USA. Continuous data were expressed as mean ± SD; categorical variables were expressed as %.

## 3. Results

A total of 34 patients with uncomplicated diverticular disease, who underwent elective segmental left colectomy, were included in the study.

Patients' and diseases' characteristics were shown in Table 1. Twenty-one patients were male, mean age was 54.1 ± 11.3, and mean BMI was 26 ± 5.5. Patients with ASA Score I were 7, with ASA II were 9, and with ASA Score III were 5.

Hinchey I diverticulitis was present in 15 patients and Hinchey II in 19 patients.

All the performed sigmoidectomy were laparoscopic and no conversion to open surgery was needed.

The patients showed a low incidence of defecation disorders, like fragmented evacuation, alternating bowel function, constipation, and minor incontinence (Table 2).

In detail, the IS questionnaire administered 6 months after surgical approach showed that the patients had an average of 5 ± 2, 2 grade of incontinence, demonstrating a good condition in terms of frequency and intensity of gas incontinence.

The CS score resulted in an average of 10 ± 3, 2 grade of constipation, demonstrating few constipation symptoms, as well as low presence of incomplete evacuation sensation and unsuccessful defecation, low pain at defecation, and a mean time spent in lavatory per attempt ranging from 5 to 10 minutes.

Furthermore, health status, evaluated by SF-36 questionnaire, was demonstrated in these patients' great physical function, role, general health, and social function, with a mean value of 86.3 ± 5, 2.

The ARM 6 months after surgery showed a normal value in terms of the anal resting pressure (47 ± 13 mmHg) and an increased volume to stimulate desire to defecate (197 ± 25 ml). The length of anal sphincter was normal compared to the reference value (37 ± 5.4 mm).

## 4. Discussion

Since its introduction in the 90s, minimally invasive surgery has gained wide popularity, and nowadays, its adoption is common in many surgical fields [12].

Furthermore, it has been widely demonstrated that the minimally invasive approach has obtained wide acceptance for the treatment of a lot of pathologic conditions of the colon and the rectum. [13–18].

Above all, the laparoscopic approach for colorectal disease in a comparison with the open approach guaranteed less postoperative pain and better recovery after surgery, as demonstrated by shorter time to bowel movements and first stools, to tolerate a solid diet and shorter length of hospital stay, and improved quality of life [19–22].

Recently, it has been demonstrated that the preservation of the Inferior Mesenteric Artery (IMA) during laparoscopic approach to sigmoidectomy is associated with a low incidence of defecation disorders, such as fragmented evacuation, alternating bowel function, constipation, and minor incontinence, providing a good quality of life after surgery [23–25].

In fact, even if the surgical technique for malignant disease is standardized and includes the high-tie ligation of the IMA, this approach could be avoided for diverticular disease, because it is not necessary to reach an oncologic radicality.

On the other hand, the dissection of the mesentery close to the bowel wall is more challenging for the absence of an avascular plane.

For this reason, we have recently designed a modified caudal-to-cranial approach to facilitate the procedure.

The advantages of dissection of the mesentery close to the bowel wall has been described for conventional sigmoidectomy [26, 27].

In a recent meta-analysis on diverticular disease, Cirocchi et al. [27] analyzed data from eight studies, including 2190 patients, divided into two groups: 837 low-tie and 1353 high-tie ligation of the IMA. The obtained results showed that there was no statistically significant difference in terms of anastomotic leakage between the low- and high-tie ligation of the IMA, even if the anastomotic leakage rate was lower in the low-tie group.

At difference, in one randomized clinical trial included in this meta-analyses, Tocchi et al. [24] showed a higher incidence of radiological and clinical anastomotic leakage in the group in which the IMA was divided.

About functional results, Masoni et al. [25], in a randomized clinical trial, demonstrated that sectioning of sigmoid arteries close to the colonic wall without sectioning of the IMA may preserve the innervation improving functional results.

In fact, after the inclusion of 107 patients in the study, randomized in two groups, Masoni et al. [25] demonstrated that the 54 patients with preserved IMA presented a statistically lower incidence of defecation disorders and less lifestyle alterations than the 53 patients with the IMA sectioned below the left colic artery.

On the other hand, in an analysis on 25 patients operated on with IMA preservation for diverticular disease, Jolivet et al. [28] demonstrated that laparoscopic sigmoid colectomy with high-tie ligation of the IMA for diverticular disease did not induce functional disorders at 6 months after surgery.

Our modified caudal-to-cranial approach to perform laparoscopic segmental left colectomy for benign disease, recently designed, has brought good results in terms of long-term functional outcomes.

In fact, evaluating defecation disorders by Anorectal Manometry and three questionnaires, we have obtained an average of 5 ± 2, 2 grade of incontinence and an average of 10 ± 3, 2 grade of constipation. This data resulted in few constipation symptoms, as well as low presence of fragmentation and unsuccessful defecation, confirming the feasibility and safety of this caudal to cranial approach with ligation of the vessels close to the colonic wall.

## 5. Conclusions

Although further studies are required to obtain definitive results, our results are encouraging to propose sigmoidectomy with low-tie ligation of the IMA as the standard procedure for the treatment of diverticular disease, and our modified surgical approach could be considered useful to facilitate the surgical approach.

## Figures and Tables

**Table 1:** Patients' and diseases' characteristics.

Patients (*N*)	34
Age (years)	54.1 ± 11.3
Gender (male)	21
BMI	26 ± 5.5
ASA score	
I	7
II	9
III	5
IV	0
Comorbidity	25
Hypertension	17
Diabetes	5
Chronic bronchitis	2
Ischemic heart disease	1
Hinchey score	
I	15
II	19
III	0
IV	0

**Table 2:** Long-term functional outcomes.

Outcomes	
IS score	5 ± 2, 2
CS score	6 ± 3, 1
Anal resting pressure (mmHg)	47 ± 13
Threshold for desire to defecate (mL)	197 ± 25
Length of anal sphincter (mm)	37 ± 5.4
Health status (SF-36)	86.3 ± 5, 2

## Data Availability

Data used to support the findings of this study are available from the corresponding author upon request.

## Abbreviations

IMA: Inferior mesenteric artery
BMI: Body mass index
CT: Computed tomography
IS: Icontinence score
CS: Constipation score
ARM: Anorectal manometry.
